# Feasibility of continuous epidural analgesia in patients with failed back surgery syndrome and spinal stenosis

**DOI:** 10.1007/s00540-022-03039-6

**Published:** 2022-01-19

**Authors:** Vincent J. Heck, Bastian Himpe, Paul Kessler, Asem Almajali, Tobias Prasse, Sven Schmidt, Michael Rauschmann

**Affiliations:** 1 Orthopedic University Hospital Friedrichsheim, University Hospital Frankfurt, Marienburgstrasse 2, 60528 Frankfurt am Main, Germany; 2 Faculty of Medicine and University Hospital Cologne, Department of Orthopedics and Trauma Surgery, University of Cologne, Kerpener Str. 62, 50937 Cologne, Germany; 3 Center for Spinal Surgery, St. Elisabethen-Krankenhaus Frankfurt, Ginnheimer Straße 3, 60487 Frankfurt am Main, Germany; 4 Department of Anesthesiology, Intensive-Care Medicine and Pain Therapy, University Hospital of Frankfurt, Theodor-Stern-Kai 7, 60590 Frankfurt am Main, Germany; 5 King Hussein Medical Center, King Abdullah II St 230, Amman, 11733 Jordan; 6 Center for Spinal Surgery, Sana Klinikum Offenbach, Starkenburgring 66, 63069 Offenbach, Germany

**Keywords:** Failed back surgery, Spinal stenosis, Epidural block, Epidural analgesia, Epidural catheter

## Abstract

**Purpose:**

The purpose of this study was to outline the feasibility of continuous epidural analgesia in the treatment of failed back surgery syndrome (FBSS) or spinal stenosis.

**Methods:**

We queried our prospective collected institutional database to include all consecutive patients, who underwent continuous epidural analgesia with accompanying intensive physiotherapeutic exercise within a timeframe of 4 years. Patients suffered from FBSS or spinal stenosis; protocolled continuous epidural analgesia was planned for 4 days within the framework of an inpatient multimodal pain therapy concept. The instillation technique of the epidural catheter, the capability to attend in accompanying physiotherapy, and the peri-interventional complications were evaluated.

**Results:**

153 patients with an average age of 57.4 years (± 11.9) were enrolled in this study. 105 patients suffered from FBSS and 48 patients had spinal stenosis. Overall, 148 patients (96.7%) reported the pain reduction and were able to perform daily intensified physiotherapeutic exercise. There were no serious adverse events, neither infection nor bleeding, no cardiopulmonary complication or permanent neurological deficits. The most common side effect was neurological impairment, such as numbness, dysesthesia, or weakness of the lower limbs with complete regression after flow rate adjustment. Patients with FBSS were more likely to develop dysesthesia (*p* = 0.007).

**Conclusions:**

Continuous epidural analgesia is feasible in patients with FBSS or spinal stenosis. This treatment enables extensive physiotherapeutic treatment even in patients with severe pain conditions and can be considered as an alternative to epidural injections. An increased complication rate in comparison to short-term perioperative or perinatal application was not observed.

## Introduction

There has been an increasing rate of surgery for degenerative spinal diseases within the last 30 years due to wider availability of radiological imaging for the broader public and improvement of surgical techniques toward novel minimal invasive and reconstructive procedures [1]. Nevertheless, conservative treatment is still inevitable, especially in the treatment of multimorbid patients and patients suffering from postoperative or chronic pain. For example, in failed back surgery syndrome (FBSS), surgical treatment does not yield promising results with insufficient pain release in up to 70% of the patients undergoing revision surgery [2].

Local instillation of anesthesia yields promising results in patients suffering from FBSS or spinal stenosis [3, 4]. Due to the nature of infiltrations, the infiltration of a sustaining and sufficient dose of anesthesia, leading to pain release but not incapacitating the patient, is hard to achieve—especially over multiple days. Repeated epidural injections, as recommended by some authors, have a higher risk of complications and may not be tolerated by some patients [5, 6].

No study has been published yet that investigates the feasibility of continuous epidural analgesia for pain management in patients with FBSS or spinal stenosis. According to that, our study aims to evaluate the feasibility of continuous epidural analgesia in the treatment of patients with FBSS or spinal stenosis regarding three main questions:

1. Is it feasible to routinely place epidural catheters in patients with spinal pathologies, such as FBSS or spinal stenosis?
2. Does continuous epidural analgesia enable the patients to undergo intensified physiotherapeutic treatment, which was not possible before the catheter placement due to chronic low back pain? Overall, is it feasible to undergo physiotherapy with a placed epidural catheter?
3. What is the complication rate in this special cohort of patients in comparison to perioperative or perinatal application reported in the literature? Do serious adverse events occur and what are the main complications and problems following epidural catheter placement in these degenerative and post-operative spinal conditions?

## Methods

This retrospective study was conducted after approval by the ethics committee of the medical university center of Frankfurt / Main, Germany. A prospectively collected institutional database was reviewed retrospectively. All patients with chronic low back pain due to FBSS or spinal stenosis who received continuous epidural analgesia as part of an inpatient multidisciplinary biopsychosocial rehabilitation program within a timeframe of 4 years were enrolled in this study. Chronic low back pain was defined as pain which is localized between the costal margin and the inferior gluteal folds with or without radiculopathy for at least 3 months. In addition, FBSS is defined as a collective of patients with symptomatic recurrent lumbar disk herniation, insufficient pain relief, or pain recurrence after mono- or bi-segmental decompression of the lumbar spine or after mono- or bi-segmental lumbar interbody fusion. Spinal stenosis is defined as an abnormal narrowing of the lumbar spinal canal with a limitation of the pain-free walking distance due to pain or motorical dysfunction in both legs.

Inclusion criteria for this therapy were chronic low back pain due to FBSS or spinal stenosis that could not be managed by outpatient treatment and the patient’s wish for extended conservative therapy. None of the patients was able to participate in physiotherapy on a regular basis despite the existing pain medication and additionally fulfilled at least two of the following characteristics:

- (a) Severe impairment of quality of life or ability to work;
- (b) Failure of previous monomodal pain therapy;
- (c) Opioid addiction or misuse;
- (d) Accompanying psychological diseases.

All patients included in this therapy concept received continuous epidural analgesia. Single or repetitive epidural injections were not performed. Physiotherapeutic treatment included structured and repetitive exercise interventions, such as exercises for muscle strengthening, core stability, coordination and stretching muscles. Passive interventions, such as manual therapy, massage, or electrotherapy, were reduced to a minimum. Physiotherapeutic treatment was performed in individual training or in group training sessions for at least 45 to 60 min each session and at least twice a day. Exclusion criteria included (sub-) acute fractures, pregnancy, active malignoma, infections, and patients receiving full anticoagulation treatment.

### Epidural catheter placement

After patient information and obtained written consent, epidural catheter placement was performed by a senior anesthesiologist at the interspace closest to the clinical level of pathology or one segment higher or lower. For patients with FBSS the catheter was inserted above or below the surgical scar. All patients were placed in a sitting position. After disinfection of the puncture site, the Tuohy needle was placed according to the landmarks with ultrasound assistance. Using the saline loss-of-resistance technique, an epidural catheter was inserted through an 18-gauge Tuohy needle (continuous epidural set, B. Braun™ Melsungen, Germany) and advanced 3 cm beyond the needle tip. Correct position of the catheter was confirmed by a test dose of 2 ml of 0.2% ropivacaine, followed by a patient-controlled epidural analgesia (PCEA) with a background infusion of 1.5 ml/h 0.2% ropivacaine and a 3 ml bolus with a lockout time of 120 min. The flow rate was adjusted based on the pain reduction of the patient in steps of 0.1 to 0.2 ml/h until the pain reduction enabled a sufficient mobilization. In case of the occurrence of neurologic deficits, the flow rate was stopped until the patients showed a complete symptom relief. The flow rate was then restarted at 1.0 ml/h. The catheter was fixated with adhesive bandage. First mobilization was carried out under medical supervision. Complication assessment was carried out on a daily base and the flow rate adjusted if necessary. Before catheter removal triamcinolone 40 mg was applied. In case of persistent lower extremity neurologic deficits after adjusting the flow rate for more than two times or if there were signs of superficial infection or other serious side effects or in case of patient dissatisfaction the catheter was removed immediately and without corticosteroid application.

### Data collection and data analysis

Demographic and descriptive data, such as gender, age, body mass index (BMI), American Society of Anesthesiologists (ASA) score, comorbidities, duration of catheter insertion, and peri-interventional complications, were collected. In case of premature termination of the epidural catheter treatment or if physical exercise was not performed, the underlying reasons were noted. For numerical data, mean values and standard deviations or medians and quartiles are calculated depending on distribution. Categorical data are presented as absolute and relative frequencies. The parameters were compared between the two groups. The student’s *t* test was used for normally distributed data, the Wilcoxon and Mann–Whitney *U* test for non-normally distributed data and the Fisher two-sided exact test for dichotomous data and small sample sizes. A *p* value ≤ 0.05 was considered statistically significant. Statistical analysis was done using “BiAS for Windows” (version 11.09.).

## Results

### Patient demographics and clinical data

Patient characteristics and pre-existing conditions are shown in Tables 1 and 2. Overall, 153 patients (88 female, 65 male) were enrolled in this study. The average age was 57.4 years (± 11.9) and the body mass index (BMI) was 29.6 kg/m^2^ (± 6.0). 17 patients (11.1%) had an ASA-score of I, 100 patients (65.4%) were ASA II, and 36 patients (23.5%) were ASA III. The most common observed secondary diagnosis was hypertension (*n*: 60; 42%), followed by depression (*n*: 60; 39%) and neurological deficit of the lower extremity (*n*: 52; 34.0%). Patients with spinal stenosis were older (61.0 years (± 12.5) vs. 52.6 (± 10.9), *p* value < 0.001), had a higher BMI (32.0 kg/m^2^ (± 6.5) vs. 28.5 kg/m^2^ (± 5.8), *p* value: 0.001), and were more likely to suffer from hypertension (56.3 vs. 36.2%, *p* value: 0.031).

**Table 1:** Patient characteristics

Patient characteristics	All (*n*: 153)	FBSS (*n*: 105)	Spinal stenosis (*n*: 48)	*p* value
Age [year] (SD)	57.4 (± 11.9)	52.6 (± 10.9)	61.0 (± 12.5)	< 0.001*
Female	88 (57.7%)	60 (57.1%)	28 (58.3%)	> 0.999
BMI [kg/m^2^] (SD)	29.6 ± 6	28.5 (± 5.8)	32.0 (± 6.5)	0.001*
ASA-score				< 0.001*
I	17 (11.1%)	15 (14.3%)	2 (4.2%)	
II	100 (65.4%)	73 (69.5%)	27 (56.3%)	
III	36 (23.5%)	17 (16.2%)	19 (39.6%)	
Pain therapy according WHO level scheme				0.280
0	7 (4.6%)	4 (3.8%)	3 (6.3%)	
I	81 (53.0%)	53 (50.5%)	28 (58.3%)	
II	50 (32.7%)	38 (36.2%)	12 (25.0%)	
III	15 (9.8%)	10 (9.5%)	5 (10.4%)	

*SD* Standard Deviation, *ASA* American Society of Anesthesiology (score), *BMI* Body Mass index, *FBSS* Failed Back Surgery Syndrome, *WHO* World Health Organization

*Significant (*p* < 0.05)

**Table 2:** Secondary diagnosis

Pre-existing conditions	All (*n*: 153)	FBSS (*n*: 105)	Spinal stenosis (*n*: 48)	*p* value
Hypertension	65 (42%)	38 (36.2%)	27 (56.3%)	0.031*
Cardiopulmonary disease	43 (28.1%)	27 (25.7%)	16 (33.3%)	0.436
PAOD	0	0	0	NA
Traumatological or orthopedic disease	51 (33.3%)	33 (31.4%)	18 (37.5%)	0.579
Depression	60 (39.2%)	45 (42.9%)	15 (31.3%)	0.235
Neurological disease	15 (9.8%)	13 (12.4%)	2 (4.2%)	0.196
Lower extremity neurological deficit	52 (34.0%)	41 (39.0%)	11 (22.9%)	0.077
Sensory deficit	38 (24.8%)	30 (28.6%)	8 (16.7%)	0.168
Motor deficit	28 (18.3%)	23 (21.9%)	5 (10.4%)	0.139

*PAOD Peripheral Artery Occlusive Disease, *FBSS* Failed Back Surgery Syndrome, *NA* Not Applicable

*Significant (*p* < 0.05)

Patients with FBSS or spinal stenosis did not differ significantly in terms of oral pain medication according to the WHO level scheme (*p* value: 0.280, for detailed information see Table 1).

### Physiotherapeutic treatment

**Fig. 1:**
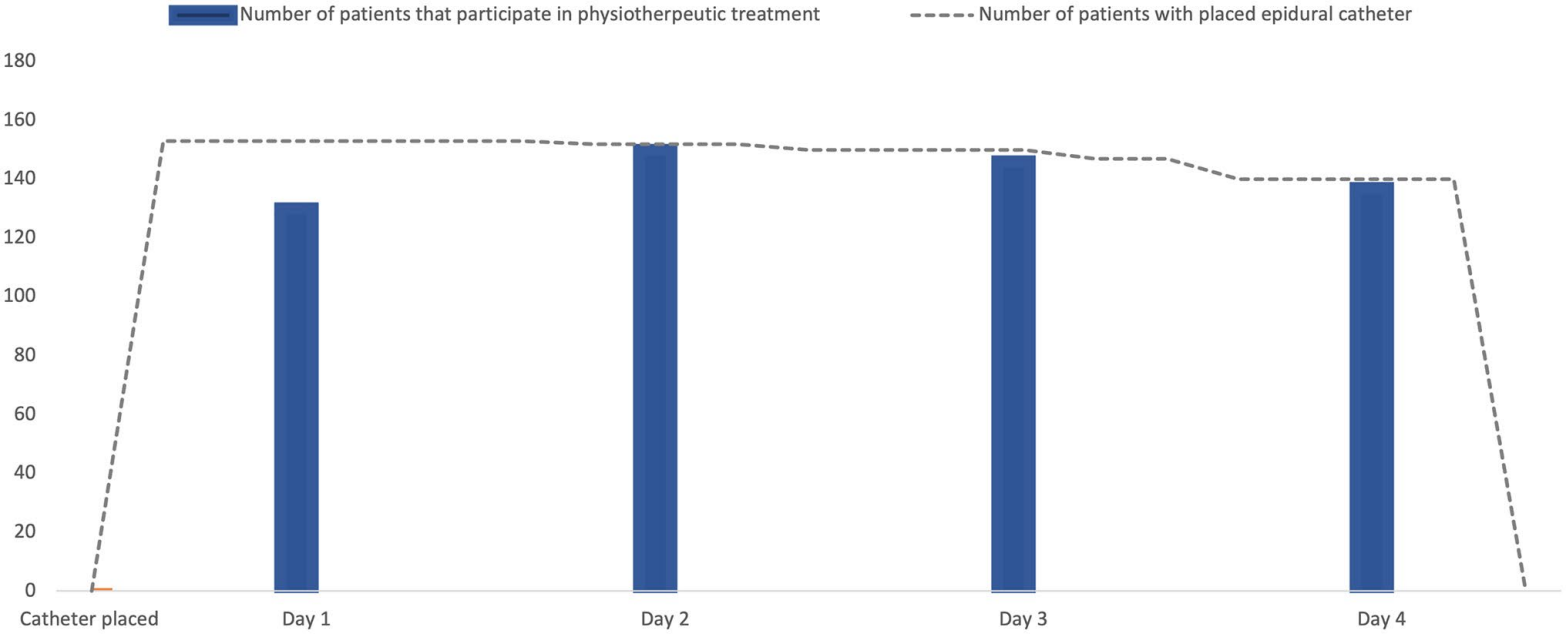
Participation in physiotherapeutic treatment with placed epidural catheter. The Y-axis shows the number of patients with placed epidural catheter (dashed line) as well as the number of patients according to their ability to participate in physiotherapeutic treatment without premature termination due to pain (blue bar). At day 1, only 128 patients participated and completed the treatment due to insufficient pain relief. At day 2, after adaption of the flow rate, 148 patients were able to fully complete the active exercises. The X-axis shows the time from catheter placement until catheter removal

Figure 1 shows the participation in physiotherapeutic treatment with placed epidural catheter. 148 out of 153 patients (96.7%) underwent regular physiotherapeutic treatment with at least two sessions per day. In three patients (2.0%), the epidural catheter treatment did not provide sufficient pain relief and therefore was removed due to patient dissatisfaction. In two patients (1.3%), the adjustment of the flow rate was difficult and recurrent lower extremity neurologic deficit occurred. Epidural catheter treatment was terminated prematurely in 13 patients (8.5%). This was mainly due to accidental dislocation in seven of 13 patients (53.8%) and patient dissatisfaction in three of 13 patients (23.1%). Groups did not differ significantly (Table 3).

**Table 3:** Premature termination of continuous epidural analgesia with reason

Premature termination	All (*n*: 153)	FBSS (*n*: 105)	Spinal stenosis (*n*: 48)	*p* value
Day 2	3 (2.0%)	3 (2.9%)	0	0.552
Day 3	10 (6.5%)	8 (7.6%)	2 (4.2%)	0.726
Reason				
Recurrent lower extremity neurologic deficit	2 (3.2%)	2 (1.9%)	0	> 0.999
Accidental pull out	7 (4.6%)	6 (5.7%)	1 (2.1%)	0.434
Patient dissatisfaction	3 (2.0%)	2 (1.9%)	1 (2.1%)	> 0.999
Others	1 (< 1.0%)	1 (< 1.0%)	0	> 0.999

*FBSS* Failed Back Surgery Syndrome

### Peri-interventional complications

No serious adverse events, no sign of cardiopulmonary affection, no dural or vascular misapplication, and no sign of epidural hematoma or abscess or allergic reaction within the study population were observed. One patient with spinal stenosis developed a temporary bladder-colon dysfunction at the time of placement. Symptoms were fully regressive after few hours. Two patients (1.3%) slipped during physiotherapy. They did not suffer from sensorimotor deficits. In no case epidural catheter treatment caused an extended inpatient stay, need for medication or for surgical intervention (Table 4).

**Table 4:** Adverse events

Adverse events	All (*n*: 153)	FBSS (*n*: 105)	Spinal stenosis (*n*: 48)	*p* value
Serious adverse events	0	0	0	NA
Moderate adverse events				
Temporary bladder-colon disturbances	1 (< 1.0%)	1 (1.0%)	0	> 0.999
Subcutaneous bleeding	0	0	0	NA
Accidental fall	2 (1.3%)	1 (1.0%)	1 (2.1%)	> 0.530
Irritation/ reddening at puncture site	4 (2.6%)	3 (2.9%)	1 (2.1%)	> 0.999
Mild adverse events / side effects				
Temporary lower extremity neurological impairment	91 (59.5%)	64 (61.0%)	27 (56.3%)	0.710
Dysesthesia	22 (14.4%)	21 (20.0%)	1 (2.1%)	0.007*
Numbness	70 (45.8%)	47 (44.8%)	23 (47.9%)	0.850
Motor deficit	15 (9.8%)	11 (10.5%)	4 (8.3%)	0.904
Reaction of the autonomic nervous system	6 (3.9%)	5 (4.8%)	1 (2.1%)	0.731
Nausea	2 (1.3%)	1 (1.0%)	1 (2.1%)	0.530
Dizziness	3 (2.0%)	3 (2.9%)	0	0.552
Headache	1 (< 1%)	1 (1.0%)	0	> 0.999
Temporary local pain	6 (3.9%)	5 (4.8%)	1 (2.1%)	0.666
Prolonged superficial bleeding	19 (12.4%)	15 (14.3%)	4 (8.3%)	0.440

*FBSS* Failed Back Surgery Syndrome, *NA* Not Applicable

*Significant (*p* < 0.05)

The most common side effect was temporary neurological impairment in 91 patients (59.5%). This included numbness in 70 patients (45.8%), dysesthesia in 22 patients (14.4%), and motor deficits in 15 patients (9.8%). Patients with FBSS were more likely to develop dysesthesia (2.1 vs. 20.0%, *p* value: 0.007). Overall, the rate of temporary lower extremity neurological impairment did not differ significantly (*p* value: 0.710). A dysregulation of the autonomic nervous system including nausea, dizziness, or headache, was rarely observed (*n*: 6; 3.9%). All these symptoms completely disappeared after adjustment of the flow rate. Eleven patients (7.2%) developed temporary local pain. 19 patients (12.4%) had prolonged bleeding at the puncture site.

## Discussion

The results of this study show that inpatient continuous epidural analgesia is feasible in patients with FBSS or spinal stenosis with a broad variety of secondary diagnoses. 148 out of 153 patients (96.7%) underwent physiotherapeutic treatment. Only in five patients (3.3%), the epidural catheter treatment did not provide pain relief or was followed by recurrent lower extremity neurologic deficit leading to no participation in physiotherapy.

In chronic low back pain, non-pharmacological approaches, such as physiotherapeutic and psychological treatment modalities, are recommended as a first-line therapy [7]. Nevertheless, multimodal analgesia or regional anesthesia often become necessary to enable sufficient mobilization of the patients. For pharmacological treatment options a Cochrane review found selective and non-selective NSAIDs to be similar in terms of effectiveness when it comes to the treatment of chronic low back pain [8]. Opioids provide the most effective pain reduction for both neuropathic and non-neuropathic pain conditions [9]. However, among other side effects NSAIDs lead to significant gastrointestinal adverse events in up to 2% of the patients [10]. On the other hand, opioids—as last-line treatment option as part of multimodal analgesia—only showed short-term pain relief in chronic low back pain. Additionally, in consideration of the side effects, opioids should only be used in certain selected patients [11]. Recently, in a prospective randomized study, Schneider et al. found manual therapy combined with individual exercise to be even more effective than medical care alone [12]. Yet, in case of insufficient pain relief with oral medication, patients were offered the possibility to receive additional epidural injections. There is broad evidence for the effectiveness of epidural injections in case of spinal stenosis and low back pain regarding the short-term outcome [10]. Because of this, in case of insufficient pain relief with oral pain medication alone, epidural injections are widely used to reduce the pain and to improve the patient’s mobilization during the inpatient multidisciplinary biopsychosocial rehabilitation program. Since single injections often provide only short-term pain relief, multiple injections can be performed to improve the pain reduction [5, 7, 10]. Therefore, a one-stage catheter placement without trial single injection is a more practical and time efficient procedure than repetitive epidural injections and reduces the probability of accidental dural puncture which is about 0.8% in lumbar interlaminar epidural injection [13]. In addition, epidural catheter use provides continuous analgesia for the entire time of the treatment and yields the possibility of dose adjustment.

Epidural catheters are widely used for peripartal and perioperative pain management, but there are only few reports with small case numbers and uncommon methodological approaches assessing the use of continuous epidural analgesia in patients with chronic low back pain [14–17]. Dolin et al. reported 46 patients who underwent 71 h of epidural analgesia on average each. All patients had chronic low back pain and participated in physiotherapy during the treatment. There was no systematic evaluation of adverse events or side effects [15]. Raj et al. compared pain relief in 15 patients with postoperative pain due to lower extremity surgery to 15 patients suffering from chronic low back pain. Both groups underwent continuous epidural anesthesia for 64 h [16]. Pain relief ranged between 55 and 96% measured by visual analogue scale. Overall, 11 of 15 patients with chronic low back pain had a urinary dysfunction, 15 patients suffered from sensory block over six to eight dermatomes and two patients showed complete motor block. Accompanying physiotherapeutic exercise was not done.

For decades, there has been an ongoing discussion on the possibility of degenerative changes of the lumbar spine such as spinal stenosis to worsen or to cause neuraxial injuries following lumbar epidural analgesia [18]. There are reports showing an association between spinal pathologies such as, spinal stenosis or lumbar disk disease, and a higher incidence of epidural hematomas and neuraxial injuries in epidural analgesia [19–21]. However, overall complication rate is low in epidural injection and in prolonged catheter use [22]. Pitkänen et al. reported 13 symptomatic epidural hematomas after performing 1.4 million neuraxial blocks in Finland from 2000 to 2009 [21]. Four of them occurred in patients with spinal stenosis. Three epidural hematomas were due to excessive doses of low molecular weight heparins; in six patients the guidelines for epidural catheter implantation did not apply. A review of literature of Neal et al. focused on the pathophysiology of spinal cord injuries after regional anesthesia, including the use of epidural catheters. Even in the setting of spinal stenosis, neuraxial injuries were found exceedingly rare [20]. Evidence was found for a possible association between spinal stenosis and a higher complication rate after neuraxial blockade. But in most cases, the spinal stenosis was not diagnosed before the intervention and was detected only as part of the complications. To summarize, there is no clear evidence that spinal stenosis or postoperative changes of the lumbar spine per se cause these higher complication rates [20].

However, catheter positioning in patients with known FBSS or spinal stenosis is challenging due to a reduced spinal canal cross-sectional area, distorted anatomy, and possible scar tissue. Thus, epidural catheter placement should only be performed by experienced anesthesiologists. Because of the retrospective nature of this study, it was not possible to document the concrete number of patients who have not received continuous epidural analgesia due to technical difficulties. Nevertheless, in our experience it is possible to routinely place epidural catheters in patients with FBSS or spinal stenosis that technically could also be treated with epidural injections.

In our study’s cohort, serious adverse events were not observed [23]. Irritation and reddening of the skin at puncture site in four patients (2.6%) with spontaneous recurrence of symptoms after catheter removal were observed. No patient developed any sign of systemic infection. Bomberg et al. retrospectively assessed the time-dependence of epidural catheter-related infection risk in 20,452 patients following surgical procedures including general, orthopedic, trauma, gynecologic or genitourinary surgery, [24]. The estimated risk for infection is 1% at day four but increased over time up to 7% at day seven and 35% at day 15. Those high risks rates were not observed in our study. This can be due to the different patient populations (patients undergo surgery vs. patients in conservative treatment).

Two patients accidentally slipped during physiotherapy. This was not secondary to sensorimotor deficits. The approximated probability of accidental falling in general population over 65 years is estimated at 0.1% per day and is further increased in patients with spinal degeneration [25, 26]. However, the risk of falling caused by either sensorimotor deficits or a vasovagal reaction remains one of the main concerns during epidural catheter treatment.

This study was limited due to its retrospective study design. There was no randomization and no further comparison with a placebo group. It would certainly be desirable to investigate the advantages and disadvantages of epidural catheter treatment compared to single or multiple epidural injections. It should also be investigated whether epidural catheter treatment can be used as an alternative to percutaneous adhesiolysis which is effective in FBSS after the failure of conservative treatment, including epidural injections [2, 27, 28].

## Conclusion

This is the first study to investigate the feasibility of continuous epidural analgesia as part of an inpatient treatment program. Continuous epidural analgesia is feasible in patients with FBSS or spinal stenosis and enables enhanced physiotherapeutic treatment even in patients with severe pain conditions. Despite the degenerative or postoperative changes of the lumbar spinal canal, an increased complication rate in comparison to perioperative or perinatal application as reported in the literature was not observed. This treatment can be considered as an alternative to conventional epidural injections and should be established in studies for different lumbar pain conditions, especially in the management of acute lumbar disk herniation prior to microdiscectomy.
